# No Evidence That Genetic Variation at the Klotho Locus Is Associated With Longevity in Caucasians from the Newcastle 85+ Study and the UK Biobank

**DOI:** 10.1093/gerona/glab361

**Published:** 2021-12-05

**Authors:** Hasnat A Amin, Heather J Cordell, Carmen Martin-Ruiz, Louise Robinson, Tom Kirkwood, Alexandra I Blakemore, Fotios Drenos

**Affiliations:** 1 Department of Life Sciences, College of Health, Medicine and Life Sciences, Brunel University London, Uxbridge, UK; 2 Population Health Sciences Institute, Faculty of Medical Sciences, Newcastle University, International Centre for Life, Central Parkway, Newcastle upon Tyne, UK; 3 BioScreening Core Facility, Biosciences Institute, Campus for Ageing and Vitality, Newcastle University, Newcastle upon Tyne, UK; 4 Population Health Sciences Institute, Campus for Ageing and Vitality, Newcastle University, Newcastle upon Tyne, UK; 5 Ageing Research Laboratories, Campus for Ageing and Vitality, Newcastle University, Newcastle upon Tyne, UK; 6 Department of Metabolism, Digestion and Reproduction, Faculty of Medicine, Imperial College London, London, UK

**Keywords:** Human genetics, Longevity, Quantitative genetics

## Abstract

The demographics of Western populations are changing, with an increase in the proportion of older adults. There is evidence to suggest that genetic factors may influence the aging process: studying these may lead to interventions to help individuals live a longer and healthier life. Evidence from several groups indicates that Klotho (*KL*), a gene encoding a single-pass transmembrane protein that acts as an FGF23 co-receptor, may be associated with longevity and healthy aging. We aimed to explore this area further by comparing the genotype counts in 642 long-lived individuals from the Newcastle 85+ Study with 18 295 middle-aged Newcastle-based controls from the UK Biobank to test whether variants at the *KL* gene locus are over- or under-represented in older individuals. If *KL* is associated with longevity, then we would expect the genotype counts to differ between the 2 cohorts. We found that the rs2283368 CC genotype and the rs9536338 C allele, but not the *KL*-VS haplotype, were associated with reaching very old age. However, these associations did not replicate in the remainder of the UK Biobank cohort. Thus, our results do not reliably support the role of *KL* as a longevity factor.

The demographics of Western populations are changing, with an increase in the proportion of older adults. There is, thus, a need to define the factors affecting maintenance of physical and cognitive health in old age. There is evidence to suggest that genetic factors may influence the aging process (1) and studying these may lead to interventions that might help individuals live a longer and healthier life.

Evidence from several groups implies that the Klotho (*KL*) gene may be associated with longevity and healthy aging. *KL* is located on chromosome 13 in humans and encodes a single-pass transmembrane protein that acts as an FGF23 co-receptor (2–4). It was first identified in mice by Kuro-o et al. (5), who showed that decreased *kl* expression resulted in a condition resembling premature aging. Since this discovery, multiple studies have been carried out to explore the relationship between genetic variants at the *KL* gene locus and longevity. These have mostly been focused on a pair of functional genetic variants, in complete linkage disequilibrium, that result in F352V (rs9536314) and C370S (rs9527025) substitutions, first reported by Arking et al. (6), and referred to as the *KL*-VS haplotype.

Arking et al. (6) went on to report that *KL*-VS heterozygotes were more common in Bohemian Czechs aged ≥75 years than in newborn controls, and also found that *KL*-VS heterozygotes became more common with age in Ashkenazi Jews aged ≥79 years (7). Invidia et al. (8) also reported that *KL*-VS heterozygotes were more common in older Italian individuals (mean age 78 years) in comparison to younger controls (mean age 53 years).

However, other studies were not able to replicate the aforementioned longevity advantage reported in *KL*-VS heterozygotes. Arking et al. (6) were not able to replicate their findings from Bohemian Czechs in either Baltimore-based Caucasians or in Baltimore-based African Americans, though it should be noted that participants defined as long lived were only ≥65 years old as opposed to ≥75 years old. Novelli et al. (9) compared U.S. participants aged between 99 and 111 years old to controls aged <35 years and Flachsbart et al. (10) compared German centenarians to middle-aged controls (60–75 years old), but neither group found evidence of a difference in allele and/or genotype frequencies between long-lived cases and younger controls.

These conflicting reports indicate that, at a population level, the relationship between the *KL*-VS haplotype and longevity remains unclear. We aim to explore this area further by comparing long-lived individuals from the Newcastle 85+ Study with middle-aged Newcastle-based controls from the UK Biobank to test whether or not variants at the *KL* gene locus are over- or under-represented in older individuals.

## Method

### Population and Study Design

We obtained long-lived cases from the Newcastle 85 Plus (N85+) Study which, in 2006, recruited 1 042 participants born in 1921 regardless of their health status (excepting those with late-stage terminal illness), including those with cognitive impairment (for whom careful procedures were followed to secure proxy consent, where appropriate). All individuals who met the recruitment criteria and were not suffering from a terminal illness were eligible. The N85+ study was approved by the Newcastle and North Tyneside 1 research ethics committee (reference number 06/Q0905/2) (11).

We obtained middle-aged controls from the UK Biobank (UKB). UKB is a large prospective cohort study that recruited >500 000 UK residents aged between 40 and 69 years of age between 2006 and 2010. The participants provided blood, urine, and saliva samples, and underwent various physical assessments, as well as touchscreen questionnaires and verbal interviews at one of the 22 assessment centers. In addition, participants are being followed up via linkage to national cancer and death registries and to NHS health records (12). UKB participants who attended the Newcastle assessment center aged ≤65 years are referred to as Newcastle UKB participants. This work was carried out under UKB application 19968.

### Genotyping

N85+ participants were genotyped at the age of 85 years using Illumina Omni genotyping arrays. The details of the QC carried out on the genetic data from the N85+ study are available in Deelen et al. (13). In addition to this QC, all variants with an INFO score of <0.8 were excluded.

In the UKB, 488 377 individuals were genotyped for up to 812 428 variants using DNA extracted from blood samples on either the UK Biobank Axiom array (438 427 participants) or the UK BiLEVE Axiom array (49 950 participants). Variant quality control metrics were provided by UKB as described previously (14). Samples that did not have genetically determined White British ancestry were excluded. A list of related individuals was provided by UK Biobank and one individual from each related pair was excluded at random. For imputed variants, all variants with an INFO score of <0.8 were excluded.

The *KL* gene is located at 13:33590571-33640282 (GRCh37.p13) and 214 variants passed QC within ±5 Kb of *KL*. These were selected for our analyses. Out of the 214 variants that were available for analyses in the UKB, 195 were available in the N85+ study after the aforementioned QC filters (see previous paragraph) were applied.

### Statistical Analyses

We used R 4.0.2 (15) to carry out analyses, unless stated otherwise. We used QCTOOL (16) and GTOOL (17) to convert both the UKB and the N85+ imputed data to hardcalls, using a posterior probability threshold of 0.9.

We used the chi-squared (χ ^2^) test to compare genotype counts between Newcastle UKB participants and N85+ participants. We used the Z test of proportions, as implemented in the prop.test(…,correct=FALSE) function in R, to compare the proportion of rs9536314 carriers and rs9536314 heterozygotes between the 2 aforementioned groups. For rs9536314, we tested 4 different genotype models (additive, dominant, recessive, and heterozygous), so the multiple-testing corrected *p*-value threshold we used was .05/4 = .0125. If the genotype distribution at any variant (except rs9536314, where all genotype models were tested regardless) was found to differ significantly between the N85+ cohort and the Newcastle UKB cohort, we identified the underlying genetic model and then used 2×2 contingency tables to compare the proportion of N85+ participants (the “event”) between the 2 genotype/allele groups (the “exposed” and “unexposed” groups). Odds ratios and the corresponding 95% confidence intervals and *p*-values were generated as described by Szumilas (18). To replicate any positive results, we separated the non-Newcastle UKB participants by the assessment center location that they attended at baseline (UKB field 54), compared the proportion of UKB participants aged ≥80 years (as of April 26, 2020) between 2 genotype/allele groups, and meta-analyzed the results using the Mantel-Haenszel method as implemented in the metabin(…,sm=“OR”,method=“MH”) function from the “meta” package in R (19).

## Results

After QC, there were 642 N85+ participants (60.6% female) and 18 295 Newcastle UKB participants (54.9% female) remaining. There are no statistically significant associations between *KL* variants and sex across the UKB (Supplementary Table 1).

Since the 2 variants making up the *KL*-VS haplotype are well-characterized functional *KL* variants in humans, we investigated whether or not their genotype distributions differed significantly between N85+ participants and Newcastle UKB participants (Table 1). We found no significant difference in the genotype distribution for rs9536314 between the 2 cohorts (TT, TG, GG: [69.5%, 27.6%, 3.0%] vs [70.4%, 27.1%, 2.5%], *p* = .74). It should be noted that only the results for rs9536314 are provided because rs9536314 and rs9527025 are in complete linkage disequilibrium (*R*^2^ = 1).

**Table 1.** Distribution of Alleles and Genotypes at rs9536314 in N85+ and Newcastle UKB (N_UKB) Participants

	N85+		N_UKB	
Genotype				
TT	446	69.5%	12880	70.4%
TG	177	27.6%	4954	27.1%
GG	19	3.0%	461	2.5%
Additive				
T	1069	83.3%	30714	83.9%
G	215	16.7%	5876	16.1%
Dominant				
TT	446	69.5%	12880	70.4%
TG/GG	196	30.5%	5415	29.6%
Recessive				
TT/TG	623	97.0%	17834	97.5%
GG	19	3.0%	461	2.5%
Heterozygous				
TT/GG	465	72.4%	13341	72.9%
TG	177	27.6%	4954	27.1%

It has been suggested that *KL*-VS heterozygotes are at an advantage when it comes to longevity. We therefore compared the proportion of rs9536314 heterozygotes between N85+ participants and Newcastle UKB participants, but found no difference (27.6% vs 27.1%, *p* = .79). We also compared the proportion of rs9536314 GG homozygotes and rs9536314 G carriers between the 2 cohorts, but again did not find any differences (3.0% vs 2.5%, *p* = .49; and 30.5% vs 29.6%, *p* = .61). The allele frequencies were also similar (16.7% vs 16.1%, *p* = .51). The results are summarized in Table 1.

We next sought to compare the genotype distributions between the N85+ participants and the Newcastle UKB participants for the remaining 194 *KL* variants (Supplementary Table 2). The genotype distributions of rs2283368 and rs9536338 differ between the 2 cohorts (*p* = 2.1 × 10^−3^ and *p* = 7.5 × 10^−3^, respectively). These variants were selected for further analysis.

**Figure 1.**
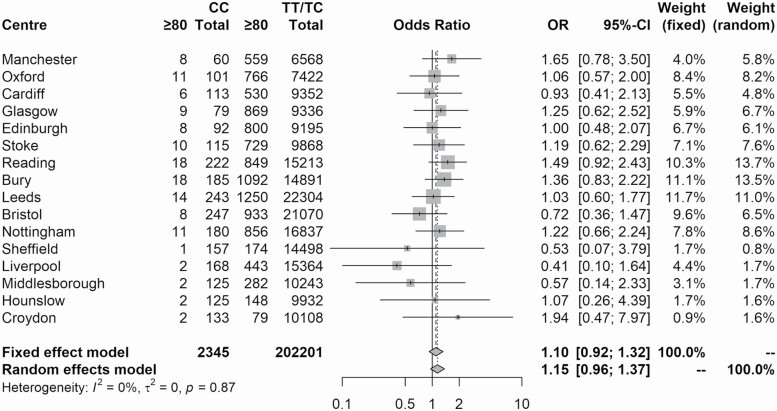
Meta-analysis of the distribution of rs2283368 CC genotypes among long-lived cases and younger controls in the UKB.

We found that individuals from the N85+ study were more likely to be present in the rarer rs2283368 CC group than the rs2283368 TT/TC group (OR = 2.42, [95% CIs 1.44–4.06, *p* = 4.0 × 10^−4^]), which suggests that the CC genotype could be associated with longevity (Table 2). However, when we attempted to replicate this result by comparing the proportion of UKB participants aged ≥80 years who were present in the rs2283368 CC group to the proportion who were present in rs2283368 TT/TC group at each assessment center across the United Kingdom (Figure 1), we found no statistically significant difference (random effects model: OR = 1.15 [95% CIs 0.96–1.37, *p* = .14]).

**Table 2.** Distribution of rs2283368 CC Genotypes Among N85+ and Newcastle UKB (N_UKB) Participants

	CC	TT/TC
N85+	16	626
N_UKB	184	17435
	200	18061

**Figure 2.**
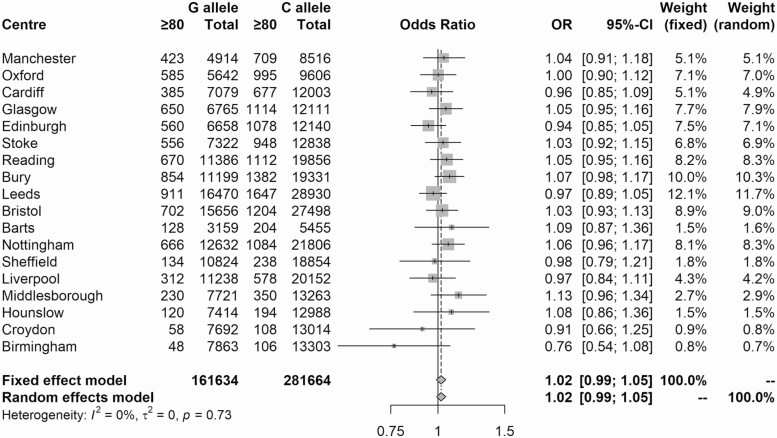
Meta-analysis of the distribution of G and C alleles at rs9536338 among long-lived cases and younger controls in the UKB.

We also found that the rarer rs9536338 G allele is less frequent in N85+ participants (OR = 0.81 [95% CIs 0.72–0.92, *p* = 6.3 × 10^−3^]), which suggests that the G allele may be associated with reduced longevity (Table 3). We attempted to replicate this finding by comparing the proportion of UKB participants aged ≥80 years who were present in the rs9536338 G group to the proportion who were present in the rs9536338 C group at each assessment center (Figure 2), but we did not find any evidence to support our initial result (random effects model: OR = 1.02 [95% CIs 0.99–1.05, *p* = .2]).

**Table 3.** Distribution of G and C Alleles at rs9536338 Among N85+ and Newcastle UKB (N_UKB) Participants

	G	C
N85+	352	768
N_UKB	12785	22697
	13137	23465

## Discussion

In this study, we sought to verify previous reports of associations between the *KL*-VS haplotype and longevity and to identify novel variants at the Klotho gene locus that may also be associated with longevity, if any. Our data do not support the presence of an association between rs9536314, a genetic variant that characterizes the *KL*-VS haplotype, and longevity. Although we identified possible associations with the rs2283368 and rs9536338 variants, we were unable to replicate these in a second, much larger, sample.

Arking et al. (6) reported an association between rs9536314 and longevity, but we could not identify this in our data. A possible reason for this may be that Arking et al. (6) compared newborns to older participants, which means that the effect they observed could be explained by a relationship between rs9536314 and infant mortality as opposed to longevity. Indeed, 2 other studies (9,10), in which adults, rather than newborns, were used as controls, also did not provide evidence for an association between longevity and rs9536314.

We were unable to replicate the associations that we found between rs2283368 and rs9536338 and longevity, so they are likely to be false positives. However, it is also possible that the lack of UKB participants aged ≥85 years and the consequent need to re-define long-lived cases as those aged ≥80 years may have reduced the power of our replication sample to detect an effect, if present.

Previous studies have used a variety of age thresholds to define their long-lived cases and their younger controls, which makes it difficult to compare them and to establish a pattern. We chose to use 85 years as the threshold to define our long-lived cases and this seems reasonable given that the pre-pandemic life expectancy in most countries, including the United Kingdom (20), has not yet exceeded 85 years and previous publications frequently consider those aged above 85 years as the oldest old (21). We defined our middle-aged controls as those aged between 45 and 64 years inclusive because this is the current MeSH definition of middle age (22). Another approach, used by Invidia et al., (8) involves generating population-specific survival curves and defining thresholds based upon the ages at which mortality increases or decreases. However, these ages are likely to vary between populations (and therefore will be subject to population-specific biases) and will be affected by events such as the COVID-19 pandemic (23).

Participants in the UKB study are reportedly healthier than the average for a person from the UK population (24). It could be argued that individuals who are likely to be long lived tend to be free of any age-related morbidities until the very end of their life (25), so the UKB sample may contain a higher proportion of individuals who will ultimately be long lived. The Newcastle UKB cohort, which was the control sample in this study, may therefore contain some individuals who, in time, would be included as cases in studies such as ours. Furthermore, the N85+ Study sought to recruit participants regardless of health status (excepting those with late-stage terminal illness), including those with cognitive impairment (for whom careful procedures were followed to secure proxy consent, where appropriate), which reduces the difference between the long-lived cases and younger controls because maintenance of cognitive independence into very old age is a characteristic of longevity (26). Together, these factors may have reduced the power of our discovery sample to detect an effect, if present.

It is also important to note that both the UKB and the Newcastle 85+ Study have a higher proportion of female participants. It is also well known that women live longer than men (27). However, since there is no association between any of the *KL* variants that we tested and sex, this is unlikely to have made a difference to our results.

In conclusion, we did not find sufficient evidence to support the previously reported associations between *KL*-VS and longevity. Once further follow-up data from the UKB become available as the cohort gets older and some individuals begin to exceed the average life span, the associations between rs2283368 and rs9536338 and longevity should be re-tested. However, despite the novel (albeit unreproduced), associations that we describe, the evidence, at least on a population genetics level, remains fragmented. Thus, our results do not reliably support the role of *KL* as a longevity factor.

## Supplementary Material

glab361_suppl_Supplementary_Tables_S1-S2
